# No difference in human mast cells derived from peanut allergic versus non‐allergic subjects

**DOI:** 10.1002/iid3.226

**Published:** 2018-07-10

**Authors:** Lau F. Larsen, Nanna Juel‐Berg, Anker Hansen, Kirsten S. Hansen, E. N. Clare Mills, Ronald van Ree, Madeleine Rådinger, Lars K. Poulsen, Bettina M. Jensen

**Affiliations:** ^1^ Allergy Clinic Copenhagen University Hospital Gentofte Copenhagen Denmark; ^2^ Medical Prognosis Institute Hoersholm Denmark; ^3^ Division of Infection, Immunity and Respiratory, School of Biological Sciences, Manchester Institute of Biotechnology Manchester Academic Health Sciences Centre, The University of Manchester Manchester UK; ^4^ Departments of Experimental Immunology and of Otorhinolaryngology Academic Medical Center, University of Amsterdam Amsterdam The Netherlands; ^5^ Department of Internal Medicine and Clinical Nutrition, Krefting Research Centre Institute of Medicine, University of Gothenburg Gothenburg Sweden

**Keywords:** Food allergy, mast cells, peanut

## Abstract

**Introduction:**

Mast cells are the primary effector cells of allergy. This study aimed at characterizing human peripheral blood‐derived mast cells (PBdMC) from peanut allergic and non‐allergic subjects by investigating whether the molecular and stimulus‐response profile of PBdMC discriminate between peanut allergic and healthy individuals.

**Methods:**

PBdMC were generated from eight peanut allergic and 10 non‐allergic subjects. The molecular profile (cell surface receptor expression) was assessed using flow cytometry. The stimulus‐response profile (histamine release induced by secretagogues, secretion of cytokines/chemokines and changes in miRNA expression following anti‐IgE activation) was carried out with histamine release test, luminex multiplex assay and miRNA arrays.

**Results:**

Expression of activating receptors (FcϵRI, CD48, CD88, CD117, and C3aR) on PBdMC was not different among peanut allergic and non‐allergic subjects. Likewise, inhibitory receptors (CD32, CD200R, CD300a, and siglec‐8) displayed comparable levels of expression. Both groups of PBdMC were unresponsive to substance P, compound 48/80 and C5a but released comparable levels of histamine when stimulated with anti‐IgE and C3a. Interestingly, among the secreted cytokines/chemokines (IL‐8, IL‐10, IL‐13, IL‐23, IL‐31, IL‐37, MCP‐1, VEGF, GM‐CSF) PBdMC from peanut allergic subjects showed a different secretion pattern of IL‐31 compared to non‐allergic subjects. Investigating miRNA expression from resting or activated PBdMC revealed no significantly difference between peanut allergic and non‐allergic subjects.

**Conclusion:**

The molecular and stimulus‐response profile revealed that PBdMC from peanut allergic subjects differently express IL‐31 compared to non‐allergic subjects. However, since only one altered parameter was found among 893 investigated, it is still questionable if the pathophysiological mechanisms of peanut allergy are revealed in PBdMC.

## Introduction

Peanut allergy is estimated to affect 0.2% of the European population, is rarely outgrown, and is the common culprit food most frequently causing a severe life‐threatening allergic reaction following exposure to trace amounts 1, 2, 3. The primary effector cell of food allergy is the IgE‐sensitized tissue‐resident mast cell, which, due to its mediator release, cause the clinical symptoms observed during a peanut allergic reaction such as oral swelling and itching, nausea, stomach cramps, urticaria, diarrhea, hives, and low blood pressure 4. Upon allergen cross‐linkage of allergen‐specific IgE bound to high affinity IgE receptors (FcϵRI), mast cells release histamine, lipid mediators, cytokines, and chemokines. Released histamine and lipid mediators induce the early allergic response considered to put the patient at risk, whereas cytokines and chemokines contribute to the late phase response and recruitment of other effector cells of the allergic inflammation (e.g. basophils and eosinophils) 5, 6.

The study of human mast cells is challenged by the difficulties of obtaining pure human mast cells. Several human mast cell lines have been developed to get a high number of human mast cells for functional studies 7, 8. Even though human mast cell lines are useful for investigating a general mast cell mechanism they cannot be used for investigating the cellular difference between allergic and non‐allergic subjects. Human mast cells can be isolated directly ex vivo from tissue, but often the yield is insufficient for extended examination and the harsh enzymatic treatment needed to disperse mast cell from tissue may affect the functional characteristic as well as having the disadvantage of tissue often being obtained from patients undergoing surgical resection due to severe illness. A more recent approach is to generate human mast cells in vitro from hematopoietic stem cell isolated from peripheral or umbilical cord blood using prolonged culturing with stem cell factor (SCF), Interleukin 3 (IL‐3) and IL‐6 9. Peripheral blood‐derived mast cells (PBdMC) resemble in vivo human mast cells with respect to morphology, metachromatic stain, FcϵRI and CD117 expression, histamine release and secretion of chemokines and cytokines upon activation 10, 11.

Human mast cell expresses many different cell surface receptors enabling mast cells to balance the response to various triggers. FcϵRI, C3aR, CD88, and CD48 are receptors belonging to the adaptive and innate immune response, respectively, and are described to result in mast cell activation followed by the release of histamine and cytokines 12, 13, 14, 15. In contrast, receptors are also found which suppress mast cell activation. These receptors act by recruiting and activating phosphatases that counteract phosphorylation in the intracellular signaling cascade. Among these inhibitory receptors are CD32, CD200R, CD300a, and siglec‐8 5, 16, 17, 18. The expression level of activating and inhibitory receptors is important for the outcome of the mast cell response. A skewed expression of these receptors might be involved in the pathophysiology of allergic diseases.

Human mast cells are known to release histamine after IgE‐mediated activation but some phenotypes of mast cells, for example, skin mast cells, also respond to secretogeous such as the anaphylatoxins complement 3a and 5a (C3a and C5a), neuropeptides (e.g. Substance P) and bioactive chemicals (e.g. Compound 48/80) 19. Furthermore, human mast cells secrete a very large panel of proinflammatory proteins upon IgE‐mediated activation permitting development and acceleration of the allergic inflammation. A unique selection of secreted proinflammatory mediators might be found from mast cells originating from allergic subjects.

Another factor which has the capability to influence which products will be released by the mast cell is microRNA (miRNA). This is a large class of small regulatory molecules capable of controlling translation of target transcripts by binding to specific messenger RNA (mRNA) molecules and repressing their translation into functional protein products 20. The role of miRNAs in mast cell biology has become an area of research in recent years but studies have mostly been conducted using mast cells derived from rodents 21. Only a few studies investigate miRNA in primary human mast cells or human mast cell lines and it is not known if mast cells from peanut allergic subjects express a unique miRNA profile 22, 23, 24.

To our knowledge, PBdMC derived from food allergic and non‐allergic subjects has never been compared previously, hence the aim of this study was to investigate if PBdMC from non‐allergic and peanut allergic subjects differ in the molecular—(cell surface receptor expression) and functional profile (histamine release, cytokine/chemokine secretion and miRNA expression).

## Methods

### Study population

Peanut allergic patients were recruited from the Allergy Clinic, Copenhagen University Hospital, Denmark based on a positive oral food challenge tests or a convincing medical history of severe immediate reaction to peanut and peanut‐specific IgE concentration above 3.5 kU_A_/l in serum. Non‐allergic participants tolerated peanut consumption and did not suffer from any known allergy. The study conforms to the standards of the declaration of Helsinki and was approved by the committees on Health Research Ethics in the Capital Region of Denmark (H‐15001321). Written informed consent was obtained from all participants. The 24 subjects included in this study were part of a cohort consisting of 25 subjects who were also included in another study 25. One subject refused to participate in this study due to anxiety to blood sampling. Six subjects were excluded from the PBdMC study due to less than 90% purity of PBdMC after seven weeks of culture. Some subjects yielded an insufficient number of PBdMC to run all assays, thus the specific number of individuals are indicated in each figure.

### Mast cell differentiation

Two hundred milliliter whole blood was drawn from each participant by venipuncture. Human mast cells were generated as previously described 9, 13. In brief, peripheral blood mononuclear cells were purified by lymphoprep™ density gradient separation (Axis‐Shield PoC, Oslo, Norway) and CD34^+^ hematopoietic stem cells were isolated from peripheral blood mononuclear cells by magnetic‐activated cell sorting using CD34 MicroBead Kit (Miltenyi Biotec, GmbH, Bergisch Gladbach, Germany) according to the manufacturer's instruction. Enriched CD34^+^ cells were cultured in StemPro®‐34 medium (Invitrogen, Carlsbad, CA) supplied with 100 ng/ml recombinant human SCF (PeproTech, Rocky Hill, NJ), 100 ng/ml recombinant human IL‐6 (PeproTech), 100 U/ml penicillin (Sigma–Aldrich, St Louis, MO), 100 μg/ml streptomycin and 2 mM L‐glutamine (Sigma–Aldrich). Recombinant human IL‐3 (30 ng/ml) (PeproTech) was included in the medium for the first week only. After 7–8 weeks of culture, the PBdMC were used for experiments. The purity of cultured PBdMC was assessed by toluidine blue staining and flow cytometry analysis and revealed >90% metachromatic stained cells and CD117^+^FcϵRI^+^ cells.

### IgE sensitization and anti‐IgE stimulation of cultured PBdMC

PBdMC were sensitized with 1 μg/ml human myeloma IgE (Calbiochem, Merck Chemicals, Beeston, Nottingham, UK) per 400,000 cells for 18–24 h or left unsensitized (resting cells). PBdMC were washed twice with medium to remove unbound IgE. PBdMC were resuspended in medium (sensitized cells) or medium added 1 μg/ml goat anti‐human IgE (KPL, Gaithersburg, MD) (stimulated cells) and incubated at 37°C for 1 and 18 h. PBdMC were centrifuged at 500 g, 10 min. Supernatants were collected and stored at −80°C until luminex multiplex analysis. Cell pellets were used for flow cytometry (18 h incubated PBdMC) or stored at −80°C for miRNA arrays (1 h incubated PBdMC). For histamine release, sensitized cells were used without any further incubation.

### Flow cytometry analyses of surface receptors

#### PBdMC

Resting, IgE‐sensitized and anti‐IgE‐stimulated PBdMC were harvested and stained with Fixable Viability Dye eFluor® 780 (eBioscience, San Diego, CA) for 30 min at 4°C. PBdMC were then washed in PBS (Sigma–Aldrich) and stained with anti‐C3aR‐AF647 (BD Biosciences, Franklin Lakes, NJ), anti‐CD88‐PE‐Cy5 (Biolegend, San Diego, CA), anti‐CD117‐BV650 (BD), anti‐CD124‐AF700 (R&D Systems, Minneapolis, MN), anti‐CD126‐BV421 (BD), anti‐CD203C‐BV510 (BD), anti‐CD218a‐FITC (Biolegend), anti‐FcϵRI‐PE‐Cy7 (Biolegend), anti‐ST2‐PE (R&D Systems), and anti‐TSLPR‐BV605 (BD) or stained with anti‐CD32‐FITC (BD), anti‐CD48‐BV421 (BD), anti‐CD117‐BV650 (BD), anti‐CD172a‐PerCP‐eFlour^®^710 (ebioscience), anti‐CD200R‐PE (Biolegend), anti‐CD300a‐AF647 (Novus Biologicals, Littleton, CO), anti‐FcϵRI‐PE‐Cy7 (Biolegend) and anti‐Siglec‐8‐AF700 (R&D Systems) for 30 min at 4°C. Cells were washed and acquired on a BD Fortessa flow cytometer. Data were analyzed with FlowJo software version 10 (TreeStar, Ashland, OR). PBdMC were gated as viable CD117^+^FcϵRI^+^ cells (Fig. S1). Receptor expression was quantified using median fluorescence intensity of the specific stain subtracted that of isotype‐matched control (Δ MFI).

#### Basophils

Heparinized whole blood was drawn from all study participants. Blood was diluted 1:1 (v:v) with RPMI 1640 (Sigma–Aldrich), added anti‐C3aR‐AF647 (BD), anti‐CD3‐BV711 (BD), anti‐CD14‐APC‐eflour780 (ebioscience), anti‐CD88‐PE‐Cy5 (Biolegend), anti‐CD123‐BV650 (BD), anti‐CD124‐AF700 (R&D) anti‐CD193‐BV421 (BD) and anti‐CD294‐PE‐CF594 (BD) or stained with anti‐CD3‐BV711 (BD), anti‐CD14‐APC‐eflour780 (ebioscience), anti‐CD32‐FITC (BD), anti‐CD123‐BV650 (BD), anti‐CD172a‐PerCP‐eFlour^®^710 (ebioscience), anti‐CD193‐BV421 (BD), anti‐CD200R‐PE (Biolegend), anti‐CD300a‐AF647 (Novus) and anti‐Siglec‐8‐AF700 (R&D Systems). Blood was incubated with or without 1 μg/ml polyclonal goat anti‐human IgE (KPL) for 30 min at 37°C using a water bath. Erythrocytes were lysed with BD FACS™ lysing solution (BD). Cells were washed and stained with anti‐FcϵRI‐PE‐Cy7 (Biolegend) for 30 min at 4°C. Cells were washed, fixated using BD CellFix™ (BD) and acquired on a BD Fortessa flow cytometer. Data were analyzed as described above. Basophils were gated as CD3^‐^CD14^‐^CD193^+^CD123^+^ cells (Fig. S2A).

### Luminex multiplex

PBdMC secreted products were quantified using ProcartaPlex Mix&Match 22‐plex (ebioscience) according to the manufacturer's instruction. The assay measured; Eotaxin (49), Eotaxin‐3 (0.7), GM‐CSF (13), IL‐1beta (2.0), IL‐4 (43), IL‐5 (16), IL‐8 (8.5), IL‐10 (2.2), IL‐13 (8.5), IL‐18 (25), IL‐22 (27), IL‐23 (13), IL‐31 (55), IL‐33 (2.1), IL‐37 (15), MCP‐1 (4.7), MIP‐1α (0.8), RANTES (1.1), TNF‐α (25), TSLP (2.2), VEGF‐A (20), and VEGF‐D (1.6), numbers in parenthesis designate detection limit (pg/ml). The samples were analyzed on a Bio‐Plex 200 system using Bio‐Plex Manager 5.0 (BioRad, Berkeley, CA).

### miRNA array

PBdMC pellets were lysed in 1 ml TRIzol® reagent (Thermo Fisher Scientific, Waltham, MA), added 200 µl 1‐bromo‐2 chloropropane (BCP) (Merck KGaA, Darmstadt, Germany), and centrifuged at 15,000*g* for 15 min to separate the cell lysate in two phases. The BCP top phase containing RNA was harvested, mixed 1:1 (v:v) with phenol/chloroform (Thermo Fisher Scientific) and centrifuged 15,000 g for 15 min. The top phase was harvested, mixed with phenol/chloroform again and phase separated. This step was repeated until a distinct and debris‐free interface was visible. The top phase was harvested and washed 1:1 (v:v) in BCP to remove phenol residues. Extracted RNA was precipitated with ice‐cold isopropyl alcohol (Merck) 1:1 (v:v), centrifuged at 15,000*g* for 15 min, aspirated completely and washed in ice‐cold 80% ethanol (Merck) at 15,000*g* for 5 min. RNA pellet was air‐dried and dissolved in RNase‐free H_2_O (Thermo Fisher Scientific). Total RNA yield was determined spectrophotometrically measuring absorbance at 260 nm and relative RNA purity was determined by calculating the ratio of absorbance at 260 and 280 nm. More than 200 ng total RNA/sample was labeled with biotin‐3DNA® conjugates and run on GeneChip® miRNA 1.0 array using FlashTag™ Biotin HSR RNA Labeling Kit according to the manufactures’ instructions (Affymetrix, Santa Clara, CA). miRNA array data were analyzed using the statistical software R version 3.3.1 (R Core Team, Vienna, Austria). Using package; Affy, mirna10cdf, annotate and gplot.

### Histamine release assay

Sensitized PBdMC, kept in pipes buffer (RefLab, Copenhagen, Denmark) containing 0.5% (v:v) human serum albumin (CSL Behring GmbH, Marburg, Deutschland), were incubated for 1 h at 37°C with polyclonal goat anti‐human IgE (KPL), Substance P (Sigma–Aldrich), Compound 48/80 (Sigma–Aldrich), Complement 3a (R&D systems), Complement 5a (R&D systems) or phorbol 12‐myristate 13‐acetate + ionomycin (both from Sigma–Aldrich). PBdMC were centrifuged 300*g*, 10 min and supernatants harvested and transferred to glass fiber‐coated microtiter plates (RefLab, Copenhagen, Denmark) and incubated at 37°C for 30 min. Released histamine was detected using a fluorescence method (Reflab). In brief, plates were washed repeatedly with demineralized water, incubated with 0.4% SDS (RefLab) for 10 min and washed with demineralized water. Plates were incubated with 0.1 mg/ml o‐phthaldialdehyde (Reflab) at alkaline pH to allow formation o‐phthaldialdehyde:histamine fluorescent complexes. After 10 min the reaction was stopped by decreasing pH with 0.59% perchloric acid and the fluorescence intensity of each well were quantified on a Histareader (Reflab). The measured fluorescence intensity was converted to amount of released histamine using a standard curve. The percentage histamine release was calculated as: (released histamine of stimuli/maximum histamine release induced by phorbol 12‐myristate 13‐acetate + ionomycin stimulation) · 100.

### Statistical analysis

The statistical analysis performed is written in the legend of each figure. Significance was determined using a 2‐sided α level of 0.05. Statistical analysis was performed with GraphPad Prism 7 (GraphPad Software Inc., La Jolla, CA).

## Results

### Characteristics of the study population

Ten peanut allergic and 14 non‐allergic subjects participated in the study. Four non‐allergic and two peanut allergic individuals were excluded from the PBdMC study as these subjects did not yield pure cultures of PBdMC (<90%). The characteristics of the resulting study population are shown in Table 1. The age of the peanut allergic patients was significantly lower than the non‐allergic participants and more peanut allergic patients presented with atopic comorbidities compared to non‐allergic individuals. All peanut allergic participants had IgE to peanut and Ara h 2 above 5.4 and 4.1 kUA/l, respectively. None of the non‐allergic participants had detectable IgE to peanut and Ara h 2.

**Table 1:** Characteristics of the study population

Demographic features	Non‐allergic (*n* = 10)	Allergic (*n* = 8)	*P*‐value
Age (y)	25 (24–32)	20.5 (19–29)	**0.04** ^#^
Female sex. no. (%)	7 (70)	7 (88)	0.59
Total IgE (kU/l)	37.7 (8.8–95.7)	463 (227–3419)	**0.0014** ^#^
Specific IgE to peanut (kU/l)	<0.35‡	67.6 (18.5–90.2)	**<0.0001** ^#^
Specific IgE to Ara h 2 (kU/l)	<0.35‡	23.3 (19.3–36.0)	**<0.0001** ^#^
Other food Allergies. no (%)	0 (0)	5 (63)	**0.0065**
Urticaria. no. (%)	0 (0)	2 (25)	0.18
Atopic Eczema. no. (%)	0 (0)	8 (100)	**<0.0001**
Asthma. no. (%)	0 (0)	5 (63)	**0.0065**
Rhinoconjunctivitis. no. (%)	0 (0)	8 (100)	**<0.0001**

Values are shown as numbers (percentages) or medians (interquartile range). Statistics were done using Fisheŕs exact test or Mann–Whitney *U*‐test (#).

‡ designate below the detection limit for all non‐allergic donors.

### PBdMC from peanut allergic and non‐allergic subjects have a comparable surface receptor expression

**Figure 1:**
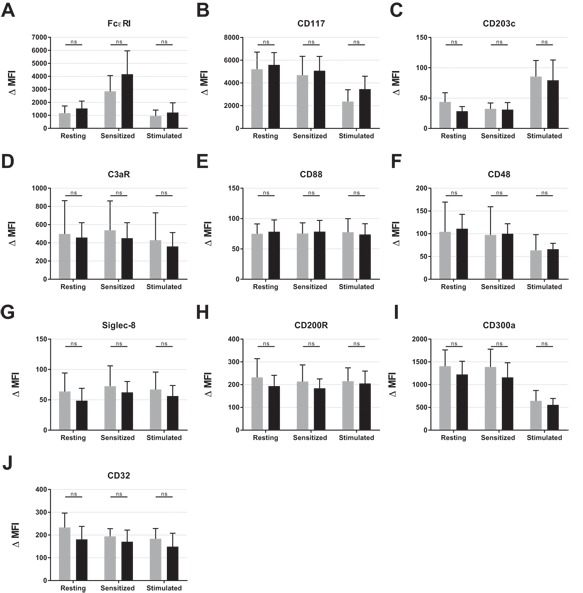
Expression of cell surface receptors on resting and activated PBdMCs from non‐allergic (*n* = 8–10) and peanut allergic (*n* = 7–8) participants. PBdMC were harvested and incubated at 37°C overnight with medium (resting cells) or medium containing human IgE to allow the cells to become sensitized. PBdMC were washed and incubated at 37°C for 18 h with (Stimulated) or without anti‐IgE (Sensitized). PBdMC were then stained with anti‐FcϵRI‐PE‐Cy7 (AER‐37(CRA‐1)), anti‐CD117‐BV650 (104D2), anti‐CD203C‐BV510 (NP4D6), anti‐C3aR‐AF647 (hC3aRZ8), anti‐CD88‐PE‐Cy5 (S5/1), anti‐CD48‐BV421 (TÜ145), anti‐Siglec‐8‐AF700 (837535), anti‐CD200R‐PE (OX‐108), anti‐CD300a‐AF647 (MEM‐260) and anti‐CD32‐FITC (FLI8.26). Statistics were performed using student *t*‐test with Bonferroni correction for multiple comparisons. Comparing non‐allergic with peanut allergic within each condition (resting, sensitized, and stimulated) did not reach statistical significance for any of the receptors. Gray bars = non‐allergic, Black bars = peanut allergic.

To examine whether receptor expression differs between PBdMC cultured from peanut allergic and non‐allergic subjects, PBdMC were left resting, sensitized with myeloma IgE, or sensitized and stimulated with anti‐IgE to mimic allergen activation. PBdMC were gated as CD117^+^FcϵRI^+^ cells (Fig. S1). Expression of CD124 (IL‐4Rα), CD126 (IL‐6Rα), CD172a (SIRPα), CD218a (IL‐18Rα), CD294 (CRTH2), thymic stromal lymphopoietin (TSLP) receptor, and ST2 (IL‐33R) were not detected on PBdMC (data not shown). The expression of mast cells specific markers FcϵRI, CD117, and CD203c did not significantly differ between peanut and non‐allergic allergic participants regardless of whether the PBdMC were resting, sensitized or stimulated (Fig. 1A–C). However, the expression of FcϵRI increased following IgE sensitization and decreased to resting levels after stimulation for both non‐allergic and peanut allergic subjects (Fig. 1A). CD117 expression decreased following activation and the activation marker CD203c was as expected elevated after anti‐IgE activation (Fig. 1B and C).

The expression of receptors known to activate mast cell and receptors able to inhibit/reduce mast cell activation were assessed to investigate whether the balance between activation and inhibition differs among non‐allergic and peanut allergic subjects. Expression of innate immune receptors capable of inducing mast cells activation (C3aR, CD88, and CD48) did not differ between the two groups (Fig. 1D–F) which was also the case for the inhibitory receptors (Siglec‐8, CD200R, and CD300a) (Fig. 1G–I). The PBdMC were also positive for CD32 (Fig. 1J) however, since the antibody used detects both CD32A, CD32B, and CD32C, where only CD32B is inhibitory, it is not possible to correlate the CD32 expression with an inhibitory capability. Lastly, a generally reduced expression of CD48 and CD300a was observed following 18 h of activation (Fig.3F and I). Overall, PBdMC cultured from non‐allergic and peanut allergic individuals display very similar receptor expression profiles.

Basophil receptor expression was quantified on the entire study population including subjects not yielding pure PBdMC cultures. Expression of TSLP receptor, ST2, CD218a, CD124, and Siglec‐8 was not detected on basophils (data not shown). Interestingly, basophils from non‐allergic and peanut‐allergic subjects also expressed similar levels of activation/potentiating receptors (CD88, CD123, CD193, CD294, and C3aR) (Fig. S2). Only FcϵRI expression was elevated in peanut‐allergic subjects compared with non‐allergic individuals. This was expected as FcϵRI expression correlates with total IgE level and the IgE level of peanut allergic subjects was significantly higher compared to non‐allergic individuals (Table 1) 26, 27, 28. Expression of inhibitory receptors on basophils (CD172a, CD200R, and CD300a) likewise did not differ between healthy and diseased (Fig. S2). All basophils were also positive for CD32 (no difference was observed between the groups) but even though healthy basophils primary express CD32B, the ratio among the three receptor subtypes might be different within the allergic subjects. All in all, activating and inhibitory receptor expression on both PBdMC and basophils did not discriminate non‐allergic from peanut‐allergic subjects.

### Histamine release is comparable between peanut allergic and non‐allergic PBdMC

**Figure 2:**
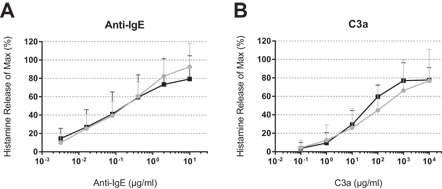
Histamine release from cultured PBdMCs derived from non‐allergic and peanut allergic participants. PBdMC from non‐allergic and peanut allergic subjects were sensitized with IgE for 18 h and stimulated with anti‐IgE or C3a for 1 h. Gray circles; non‐allergic (*n* = 10). Black squares; peanut allergic (*n* = 6–7). Plotted as mean + SD.

The degranulation capacity of PBdMC from non‐allergic and peanut allergic subjects was measured by histamine release to a selection of secretagogues. IgE‐sensitized PBdMC were activated with anti‐IgE, C3a, C5a, Substance P and Compound 48/80. Anti‐IgE and C3a activation elicited a dose‐dependent histamine release from all PBdMC and no difference was found between non‐allergic and peanut allergic subjects (Fig. 2). PBdMC did not respond with histamine release to C5a, Substance P or Compound 48/80 stimulation which the human mast cell line LAD2 did (Fig. S3).

### IL‐31 is differently expressed in PBdMC from peanut allergic subjects

**Figure 3:**
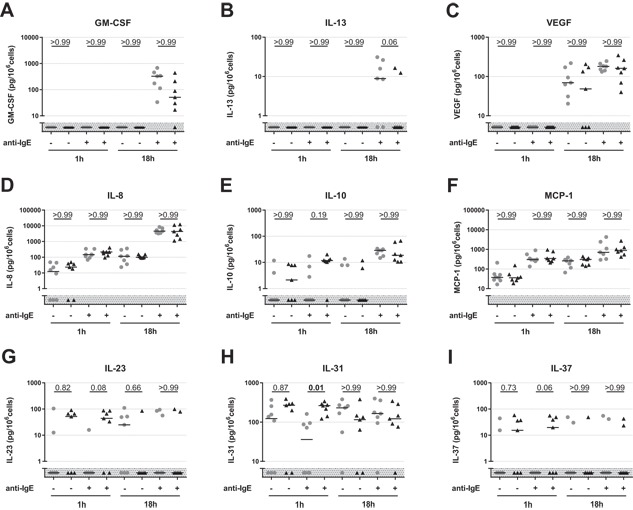
Secreted products from sensitized and anti‐IgE activated PBdMCs from non‐allergic (*n* = 7–8) and peanut allergic (*n* = 7) participants after 1 and 18 h incubation. PBdMC were harvested and incubated at 37°C overnight with human IgE to allow cells to become sensitized. PBMCs were incubated for 1 or 18 h with (+) or without (−) anti‐IgE (1 μg/ml). Collected supernatants were analyzed using multiplex bead‐based assay. Gray shaded area indicates below detection limit. Gray circles; non‐allergic subjects. Black triangles; peanut allergic patients. Black lines; medians. Statistics were performed with Dunn's multiple comparison test. *P* values are shown above each comparison.

The secretion of pre‐stored and de novo sensitized inflammatory mediators from IgE‐sensitized and anti‐IgE‐stimulated PBdMC were measured to investigate whether the cytokine/chemokine secretion profile is altered in subjects suffering from a peanut allergy. We found that PBdMC can produce GM‐CSF, IL‐8, IL‐10, IL‐13, IL‐23, IL‐31, IL‐37, MCP‐1, and VEGF (Fig. 3). PBdMC supernatant did not contain detectable levels of Eotaxin, Eotaxin‐3, IL‐1beta, IL‐4, IL‐5, IL‐18, IL‐22, IL‐33, MIP‐1α, RANTES, TNF‐α, TSLP, and VEGF‐D. In general, PBdMC secreted GM‐SCF and IL‐13 only following 18 hours of anti‐IgE stimulation indication de novo production of these cytokines upon FcϵRI activation (Fig. 3A and B). VEGF was detected in PBdMC supernatant from both sensitized and anti‐IgE‐activated cells, but only following 18 h of incubation (Fig. 3C). IL‐8, IL‐10, and MCP‐1 were continuously secreted from PBdMC and significantly augmented production was detected following mast cell activation (Fig. 3D–F). Of the nine detected cytokines, only IL‐31 was differently expressed between peanut allergic and non‐allergic subjects. IL‐31 secretion from PBdMC was significantly increased following 1 h of activation from peanut allergic subjects compared to non‐allergic individuals. However, the IL‐31 concentration changed during 18 h of stimulation, with a tendency of PBdMC from non‐allergic subjects now having the highest concentration (Fig. 3H).

### miRNA array profile does not discriminate peanut allergics from non‐allergic subjects

**Figure 4:**
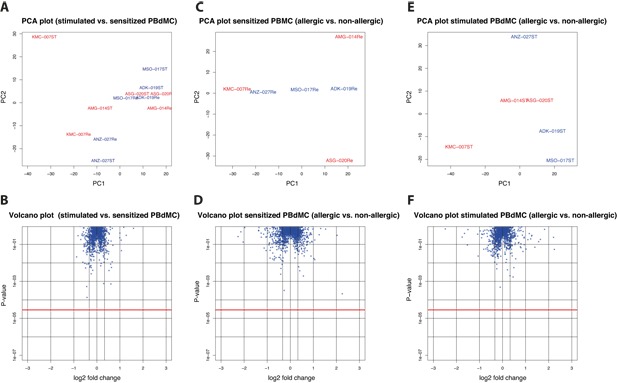
miRNA Arrays. miRNA arrays were run on resting and anti‐IgE‐stimulated PBdMCs from peanut allergic (*n* = 3) and non‐allergic (*n* = 3) subjects. A and B, PCA and Volcano plots when comparing miRNA expression between stimulated and sensitized PBdMCs. C and D, PCA and Volcano plots when comparing miRNA expression on sensitized PBdMCs between peanut allergic and non‐allergic subjects. E and F, PCA and Volcano plots when comparing miRNA expression on stimulated PBdMCs between peanut allergic and non‐allergic subjects. PCA plots, blue text: non‐allergic subjects, red text: peanut allergic individuals. Volcano plots; blue asterisks: individual miRNAs, red line: Bonferroni‐corrected statistical significant level. Statistics were performed using paired and unpaired student́s *t*‐test.

miRNA arrays, detecting 847 miRNA sequences, were performed to examine whether individual miRNAs are differently expressed in PBdMC cultured from peanut allergic and non‐allergic subjects. In general, we did not observe distinct separation of sensitized and anti‐IgE‐stimulated PBdMC with principal component analysis (PCA) or significant up‐ or down‐regulation of individual miRNA transcript following anti‐IgE activation (Fig. 4A and B). Similarly, PCA plots comparing PBdMC derived from non‐allergic and peanut allergic subjects at sensitized or stimulated state did not clearly separate the groups (Fig. 4C and E). Lastly, no statistical significantly difference was detected in individual miRNA expression between non‐allergic and peanut allergic subjects (Fig. 4D and F). Differentially expressed miRNAs with a multiple comparison non‐corrected *P*‐value below 0.01 and more than 25% difference in expression are listed in Table 2.

**Table 2:** Differentially expressed miRNAs with a non‐corrected *P*‐value <0.01 and >25% difference in expression

			Sensitized PBdMC			Stimulated PBdMC		
Stimulated vs. sensitized PBdMC			Allergic vs. Non‐allergic			Allergic vs. Non‐allergic		
miRNA	Expression change (%)	*P*‐value†	miRNA	Expression change (%)	*P*‐value†	miRNA	Expression change (%)	*P*‐value†
UP								
hsa‐miR‐132	55	1.00	hsa‐miR‐149‐3p	376	0.17	hsa‐miR‐92b‐5p	39	1.00
hsa‐miR‐557	52	0.57	hsa‐miR‐208b	28	1.00	hsa‐miR‐603	37	1.00
hsa‐miR‐1283	51	1.00						
Down								
hsa‐miR‐641	31	1.00						
hsa‐miR‐373	29	1.00						
hsa‐miR‐532‐3p	26	1.00						
hsa‐let‐7b‐3p	26	0.11						

Statistics were performed using paired and unpaired student *t*‐test.

† Bonferroni corrected *P* values.

## Discussion

We investigated whether cultured PBdMC discriminate between non‐allergic and peanut allergic subjects by examining the expression of cell surface receptors, histamine release mediated by different secretagogues, cytokine/chemokine release followed by anti‐IgE activation and miRNA expression in resting and anti‐IgE activated cells. This thorough investigation of cultured human mast cells did only reveal a molecular or functional difference in expression of IL‐31 in in vitro generated mast cells between non‐allergic and peanut allergic subjects.

PBdMC were cultured from mast cell progenitors found in the circulation. Consequently, to detect cellular differences between peanut allergic and non‐allergic subjects using in vitro differentiated PBdMC, the dissimilarities should be encoded already by the hematopoietic stem cells. Human mast cells develop and fully mature in peripheral tissue influenced by SCF and the local cytokine milieu 29. In this study SCF, IL‐6 and IL‐3 were used to develop PBdMC but additional factors may be needed to fully mature mast cells to the state found in vivo. Thus, the phenotype of our PBdMC might be different from the phenotype found in peanut allergic subjects and therefore it is possible that the mast cell phenotype could contribute to the development and the pathologic mechanisms of allergic diseases. On the other hand, ex vivo basophils from non‐allergic and peanut‐allergic subjects were only different regarding FcϵRI expression, explained by the difference in concentration of circulating IgE, thereby arguing, that the effector cells of allergy may not display a diverse cellular molecular (and functional) profile besides carrying allergen‐specific IgE on their surface. While this seems to disprove our initial proposition, it may help focus future studies on biomarkers of food allergy.

Previous studies have addressed whether PBdMC display different molecular and functional characteristics among healthy and subjects suffering from different atopic diseases. One study showed that PBdMC generated from healthy controls and patients with allergic asthma are very similar regarding degranulation measured by CD63 upregulation 30. Another study investigated PBdMC from atopic eczema patient and observed higher total histamine and tryptase levels, impaired expression of fungal recognition receptor, and enhanced IL‐6 secretion after fungal stimulation from PBdMC obtained from atopic eczema subjects compared to healthy controls. However, the release of histamine, IL‐6 and IL‐8 was not different following anti‐IgE activation 31. Thus, these studies are consistent with our overall observation i.e. secreted inflammatory mediators from progenitor‐derived mast cells are equivalent between atopic patients and healthy individuals.

In this study, we found that PBdMC released histamine upon anti‐IgE and C3a activation but not following C5a, Substance P or compound 48/80 stimulation. The lack of response to substance P and compound 48/80 deviates from previous studies on PBdMC 13, 32. The discrepancy may indicate the PBdMC generated from whole blood contra buffy coat preparation display a less mature phenotype or alternatively more resembles mast cells from lung, tonsils, and colon as these mast cells likewise do not respond to substance P and compound 48/80 19. When analyzing the released inflammatory proteins our PBdMC secreted a large variety. Interestingly, PBdMC from peanut allergic subjects seem to release more IL‐31 during 1 h but tend to have the lowest level after 18 h indicating that these PBdMC might actively take up IL‐31 over time. We did not look for IL‐31 receptor expression, which could have revealed if PBdMC from peanut allergic patients express more IL‐31R and therefore has an elevated uptake of this cytokine. It was also not possible to validate the IL‐31 production using another technical approach due to lack of test material. Nevertheless, IL‐31 is known to be secreted from PBdMC and elevated production from human mast cells has been reported in patients suffering from myeloproliferative diseases and mastocytosis 33, 34, 35. Similarly, IL‐31Rα has been detected on PBdMC by proteome analysis and is phosphorylated upon anti‐IgE‐stimulation 36. IL‐31 has been associated with pruritus as increased levels were reported in the skin of atopic dermatitis patients with severe pruritus but also induced profound itch in mice overexpressing IL‐31 37, 38. All the peanut allergic subjects included in this study had atopic eczema but no information was obtained regarding pruritus. On the other hand, it is possible that the increased expression of IL‐31 during 1 h of incubation is due to the atopic eczema present in all the allergic patients. Besides atopic dermatitis, IL‐31 has also been shown to be increased in nasal secretions following nasal allergen challenge in allergic rhinitis patients 39. However, it has not been investigated if IL‐31 is involved in food allergy. Similarly, the role of IL‐31 in mast cell biology is currently unknown. Accordingly, the difference in IL‐31 secretion among peanut allergic and non‐allergic subjects after 1 h activation observed in this study needs to be further investigated.

Among the inflammatory proteins, we did not detect in supernatant from anti‐IgE‐stimulated PBdMC, were eotaxin, IL‐5, MIP‐1α, and TNF‐α which previously have been shown to be released from PBdMC 40. Especially the absence of IL‐5 and TNF‐α was unexpected as transcription of their corresponding mRNAs do increase in PBdMC following anti‐IgE activation (unpublished results by LFL) and both cytokines have been shown to be released from ex vivo isolated human mast cells 40, 41, 42, 43. The lack of detection could be due to freeze‐thawing of the harvested supernatant or insufficient sensitivity of luminex multiplex assays used to quantify secreted protein products from PBdMC, which have previously been addressed and discussed elsewhere 13.

In conclusion, we performed a thorough molecular and functional profile of PBdMC from peanut allergic and non‐allergic subjects testing 893 parameters and only IL‐31 was found differently expressed. It is possible that in vitro differentiated PBdMC may not exhibit the cellular pathophysiological properties as of in vivo derived mast cells, but we believe it is more likely that mast cells from peanut allergic subjects are similar to healthy individuals. Allergic diseases may hence primarily be caused by specific IgE sensitization and sensitivity in the target organ towards the released mast cell mediators rather than influenced by the cellular properties of the mast cells.

## Authors' Contribution

LFL, AH, ENCM, RVR, LKP, and BMJ conceived and designed the experiments. LFL, NJB, and KSH achieved ethical approved, recruited patients and collected clinical data. LFL and AH performed the experiments. LFL, AH, BMJ, and MR analyzed the data. LFL wrote the first draft of the study. All authors contributed to revision and approved the final version of the manuscript.

## Conflicts of Interests

The authors declare that the are no conflicts of interests

## Supporting information

Additional supporting information may be found online in the Supporting Information section at the end of the article.

**Figure S1**. Flow cytometry gating on PBdMCs
**Figure S2**. Expression of cell surface receptors on resting and activated basophils from non‐allergic (n=14) and peanut allergic (n=10) participants.
**Figure S3**. Histamine release from cultured PBdMCs derived from non‐allergic and peanut allergic participants.
