# Supplementary material for: No difference in human mast cells derived from peanut allergic versus non‐allergic subjects
Source: Immun Inflamm Dis. 2018 Jul 10;6(4):416–27. doi: 10.1002/iid3.226 (PMC6247235; doi:10.1002/iid3.226)
Supplement: Supplementary file 1 — Figure S1. Flow cytometry gating on PBdMCs Figure S2. Expression of cell surface receptors on resting and activated basophils from non‐allergic (n=14) and peanut allergic (n=10) participants. Figure S3. Histamine release from cultured PBdMCs derived from non‐allergic and peanut allergic participants. [file IID3-6-416-s001.docx]

# Supplementary Figures


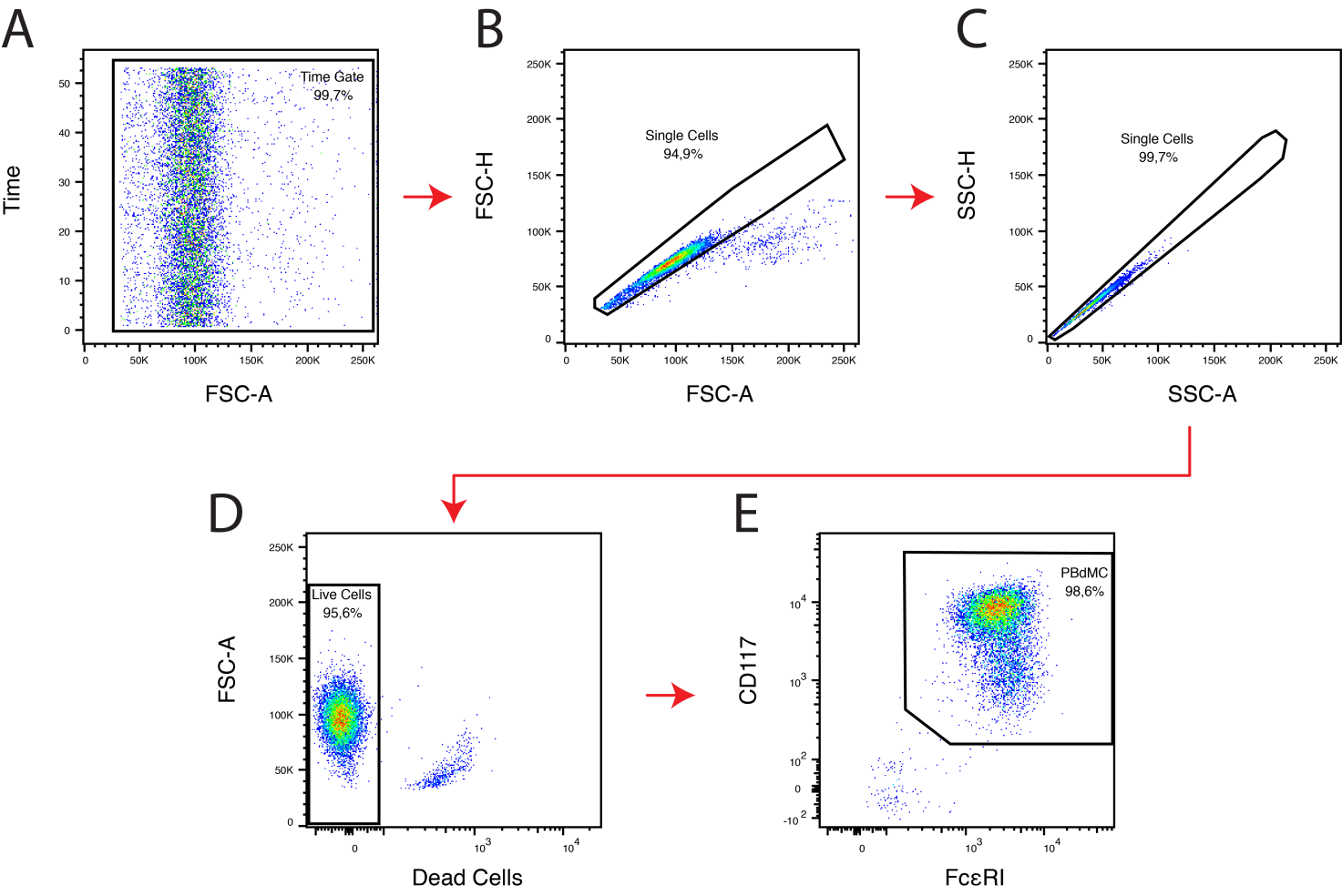


**Figure S1. Flow cytometry gating on PBdMCs.** Cells were gated on time versus forward scatter to ensure uniform flow (**A**). Single cells were selected based on forward scatter height (FSC-H) versus forward scatter area (FSC-A) and side scatter height (SSC-H) versus side scatter area (SSC-A) characteristics (**B** and **C**). Dead cells were excluded based on elevated staining with Fixable Viability Dye (**D**). PBdMCs were subsequently gated as CD117^+^FcεRI^+^ cells based on isotype-matched control (**E**).


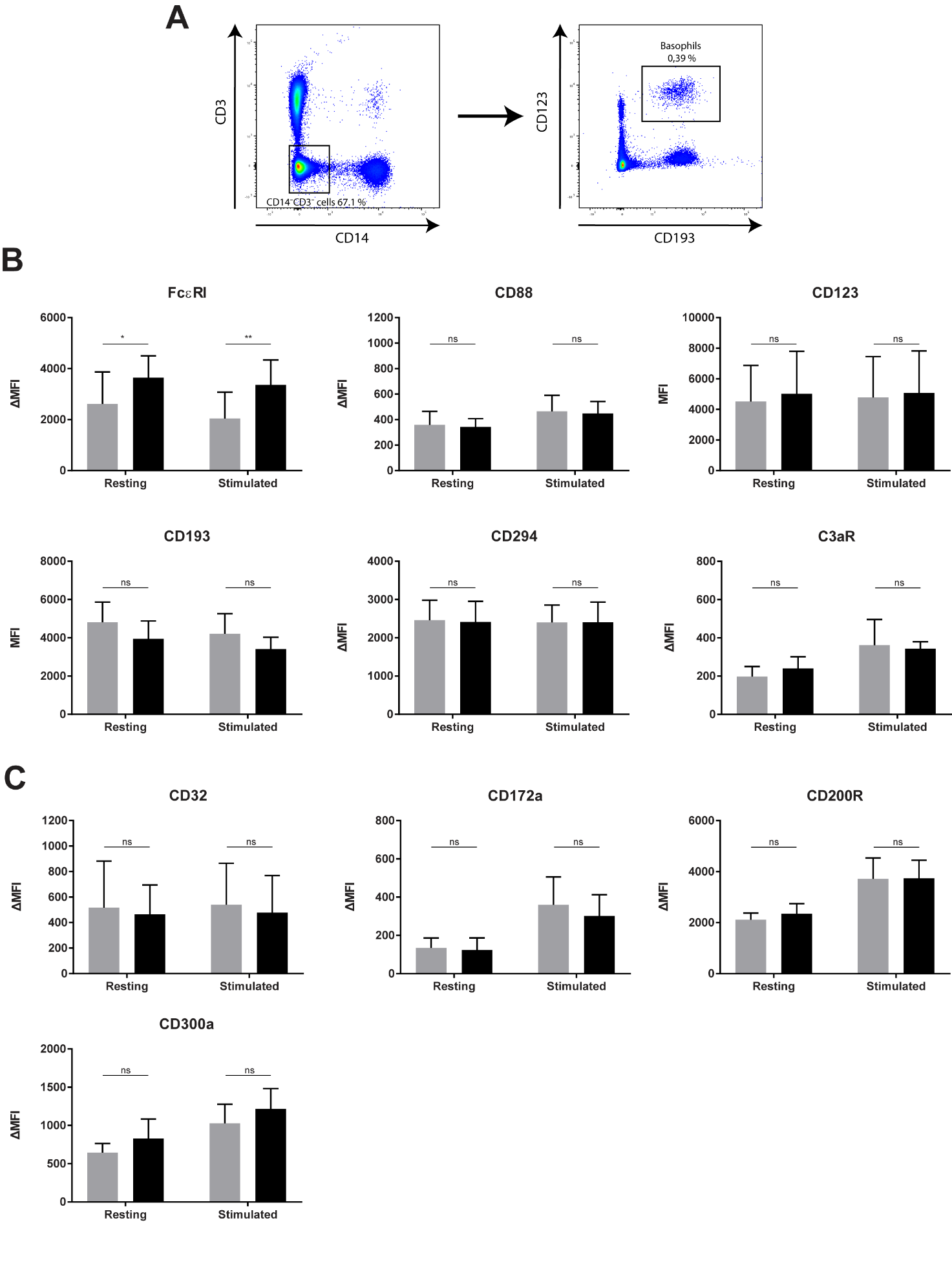
 **Figure S2. Expression of cell surface receptors on resting and activated basophils from non-allergic (n=14) and peanut allergic (n=10) participants.** Heparinized whole blood was drawn from all study participants. Blood was incubated for 30 min at 37°C with 1 µg/ml anti-IgE (stimulated) or buffer (resting) and stained with anti-CD3-BV711 (UCHT1), anti-CD14-APC-eflour780 (61D3), anti-CD32-FITC (FLI8.26), anti-CD88-PE-Cy5 (S5/1), anti-CD123-BV650 (7G3), anti-CD172a-PerCP-eFlour®710 (15-414), anti-CD193-BV421 (5E8), anti-CD200R-PE (OX-108), anti-CD294-PE-CF594 (BM16), anti-CD300a-AF647 (MEM-260), anti-C3aR-AF647 (hC3aRZ8), anti-FcεRI-PE-Cy7 (AER-37(CRA-1)). (**A**) Basophils were gated on single cells as CD3^-^CD14^-^CD123^+^CD193^+^ cells. (**B**) Expression level of basophil activation receptors. (**C**) Expression level of basophil inhibitory receptors. Statistics were performed using repeated measures two-way ANOVA with Bonferroni's post hoc multiple comparisons test. *P-value* < 0.05 (*), < 0.001 (**), ns = not significant. Grey bars = non-allergic, Black bars = peanut allergic.

**
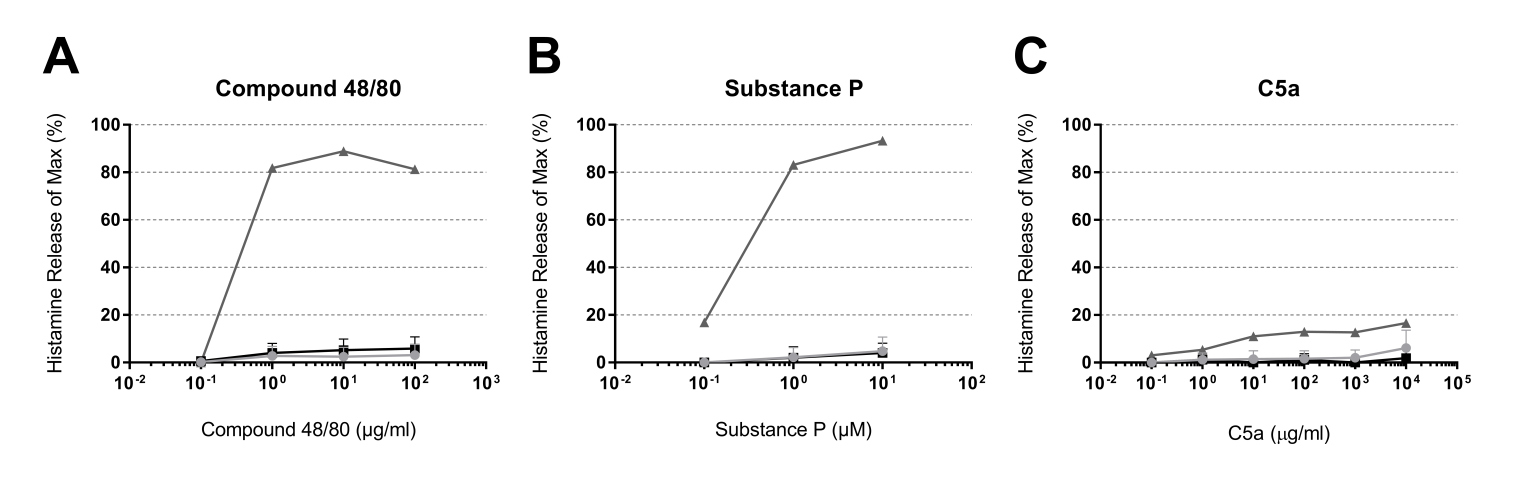
Figure S3. Histamine release from cultured PBdMCs derived from non-allergic and peanut allergic participants.** PBdMC from non-allergic and peanut allergic subjects and LAD2 cells were sensitized with IgE for 18 h and stimulated with compound 48/80, Substance P or C5a for 1 h. Non-allergic; light grey circles (*n* = 6), Peanut allergic; black squares (*n* = 6), LAD2 cells; dark grey triangles (n = 1) , plotted as mean + SD.
